# The Impact of Pandemic Perception, National Feeling, and Media Use on the Evaluation of the Performance of Different Countries in Controlling COVID-19 by Chinese Residents

**DOI:** 10.3389/fpsyg.2021.650367

**Published:** 2021-07-08

**Authors:** Ruixia Han, Jian Xu

**Affiliations:** ^1^School of Media and Communication, Shanghai Jiao Tong University, Shanghai, China; ^2^Institute of Cultural Innovation and Youth Development, Shanghai Jiao Tong University, Shanghai, China; ^3^China Institute for Urban Governance, Shanghai Jiao Tong University, Shanghai, China

**Keywords:** COVID-19, pandemic perception, national feeling temperature, media use, nation evaluation, governance

## Abstract

Different nations responded to the global spread of COVID-19 differently. How do people view the governance practices and effects of various countries? What factors affect their views? Starting from the three-dimensional model of cognitive-affective-media, this study examines how pandemic perception, the national feeling, which is the emotional preference of public for different countries, and media use affect the Chinese public views on the performance of other countries in controlling COVID-19. After performing regression analysis on the data of 619 Chinese public samples collected by an online survey, it reveals the following: (1) pandemic perception is negatively correlated with the evaluation of controlling-pandemic performance in different countries by Chinese residents, whereas national feeling is positively correlated with the evaluation of controlling-pandemic performance. (2) The use of media has different characteristics in the evaluation of controlling-pandemic performance in different countries by Chinese residents. Television has a significant influence on the evaluation of controlling-pandemic performance in the United States, China, and Germany by Chinese residents. (3) Collectivist cultural orientation has no significant impact on the evaluation of the anti-pandemic performance of different countries by Chinese residents, whereas virus perception only has a significant impact on the evaluation of the controlling-pandemic performance of the United States and Italy. Research has confirmed the existence of the cognitive-affective-media model in the evaluations by public on the governance of other countries, and prospects for the superimposed role of media in the cognitive-affective model.

## Introduction

After the outbreak of COVID-19 pandemic at the end of 2019, it has spread across the world. National governments are actively handling the virus to protect public health and social safety (Hopman et al., 2020; Kandel et al., 2020). Both traditional mainstream media and social media report dynamics about the pandemic and government responses to them. They also contain general public concerns about these controlling-pandemic measures and their effects; however, it must be noted that, while public understanding of the spread of the pandemic and the governance measures of various countries mainly comes from news media, they made their evaluation of them through social media fermentation and personal perception. In fact, the source of perception is the result of the processing of the human brain of objective reality, symbolic reality, and subjective reality, and various media has become an important medium or platform for reality construction (Adoni and Mane, 1984; Gamson et al., 1992). This role is more extensive in the era of widespread social media today. So it must be noted that there is a “mediating” or even a “filtering” effect between the formation of personal perception and real events (Feezell, 2018; Goyanes et al., 2021). In the end, this perception will become the basis of judging ability of people in face of future public health emergencies. Regarding the COVID-19 pandemic, evaluation of government ability by people in handling the pandemic is affected by the spread of the pandemic in each country, which has become the basis of scientific perception and judgment of people. At the same time, it is also affected by national feeling when it is situated in the international relations. In other words, the judgment of governance performance by people is based on the actual performance of various national governments, and they are also closely related to a national feeling and media use.

This research is trying to construct a cognitive-affective-media analysis model based on the cognitive-affective framework (Baloglu and McCleary, 1999; Goldgeier and Tetlock, 2001; Mossberg and Kleppe, 2005; Yuksel et al., 2010; Maher and Carter, 2011; Li et al., 2014), and to explore how China public evaluates the effectiveness of governance practices of other countries in social and cultural background and media environment of China. Among them, pandemic perception and national feeling are used as the main explanatory variables to examine their influence on the evaluation of governments of other countries by Chinese public. At the same time, it further analyzes how different types of media play a role in the evaluation of controlling-pandemics by other countries. Eventually, it examines the feasibility of the cognitive-affective-media analysis framework and prospects the role of the media in the formation of cognition and emotion.

## Literature Review

### Influencing Factors of Controlling-Pandemic Evaluation Under the Cognitive-Affective Model

A cognitive-affective model is common in destination image research and brand research in tourism. For example, Baloglu and McCleary (1999) pointed out that the image of a tourist destination can include three types: cognitive images, emotional images, and overall images related to them, among which cognition and emotion become the main variables that affect the destination image. To cultivate the stickiness of the destination or brand, people also put research energy into changing the cognition, emotion, and behavior of the audience (Mossberg and Kleppe, 2005; Yuksel et al., 2010). In the study of country image, people also think that there are two aspects of cognition and affection (Maher and Carter, 2011; Li et al., 2014). For the image of the country in a special (epidemic) period, we believe that the cognitive-affective model is still a very basic explanatory analysis framework. Indeed, plenty of information also shows that the perception of the pandemic and national feeling has affected the evaluation of the governance capabilities of the government by public.

Since December 2019, COVID-19 has become the global focus. The WHO regularly releases reports on the global spread of the virus, which is as important source of information for the formation of perception of the pandemic. In the meantime, however, the risk perception of residents is affected by national background. For example, the survey of Dryhurst et al. (2020) of public risk perception in 10 countries in Europe, Asia, and the America finds that individualistic worldviews, personal experience, pro-social values, and social amplification through friends and family influence public risk perception. In other words, different people have different perceptions of the spread of the pandemic in different countries based on different personal experiences and information sources. This will cause their perception of the pandemic in various countries to fluctuate based on the information released by the WHO. In summary, it is the cognitive factors of individual that will affect his evaluation of controlling-pandemic.

Since COVID-19 is an infectious virus, its spread in various countries is also closely related to the management methods of each country. From the reverse deduction of the “New Public Management” (NPM) theory (Hood, 1991) by the government, it can be directly predicted that the risk perception of COVID-19 pandemic in various countries will inevitably affect the public evaluation of the effectiveness of risk management in different countries. Indeed, many studies have objectively confirmed this result. Bodrud-Doza et al. (2020) found that the outbreak of COVID-19 created psychosocial and socio-economic insecurity in Bangladeshi citizens, which reduced trust and evaluation of the government. At the same time, some of the measures taken by governments in response to the pandemic will also affect the public evaluation of the government (Shammi et al., 2020; Sibley et al., 2020). In short, combining the perspective of personal cognition formation and the new public management theory of the government, we have reason to speculate:

H1. Public perception of the severity of the pandemic in different countries will affect their evaluation of the governance effectiveness of each country.

As early as 1964, the feeling temperature was introduced for the study of public attitudes toward prominent political groups and figures (Winter and Berinsky, 1999). Later, this term was introduced to the field of international relations studies. For example, Page et al. (2008) asked Americans to use a 0–100 scoring system to evaluate Asian countries and used an indicator system composed of personal and social characteristics, information, internationalism and domestic, antipoverty, and capitalist foreign policy goals to analyze public feeling temperature toward different countries. The emotional perception of different countries by the public will further affect their evaluation of the subsequent performance of each country. At present, most of the existing research focuses on the feeling temperature of public toward different countries and uses it as a dependent variable. However, indeed, feeling temperature can also be used as a predictive variable to influence the evaluation of government and international relations. The foundation of this influence lies in the influence of political sentiment on government evaluation. For example, as early as 1986, Lambert et al. (1986) discovered that party identity has a significant impact on the political trust by the public in the government. In recent years, further research on affective polarization has also shown the possible role of feeling temperature in political evaluation (Druckman and Levendusky, 2019). More and more people are also incorporating feeling temperature into various political prediction models (Shikano and Käppner, 2014). Combining this change with the new public management theory of the government (Hood, 1991) and the cognitive-affective model of tourism destination image (Baloglu and McCleary, 1999), we have reason to speculate:

H2. The feeling temperature of Chinese residents toward different countries will affect their evaluation of the performance of governments in managing the pandemic.

### Influencing Factor of Controlling-Pandemic Evaluation From the Perspective of Media Effects

The influence of the Chinese public on the controlling-pandemic evaluation of the governments of various countries in terms of media use mainly comes from three aspects: first, media have a priming function, i.e., the ability to isolate specific issues, events, or topics in the news so that the public pay special attention (Iyengar and Kinder, 1987). This priming effect also plays a role in the evaluation of political performance. For example, Miller and Krosnick (2000) found that when evaluating political performance of the president, media played a role in urging the public to pay attention to specific aspects of the work of the president. The “priming” function of media can even change government evaluation standards by people (Iyengar and Kinder, 1987). By drawing attention of people to certain issues, media can create, strengthen, and eliminate political judgment standards by people (McGraw and Ling, 2003). This priming effect of media also occurs in the evaluation of attitudes toward other countries. For example, Brewer et al. (2003) surveyed 199 students on the East Coast of the United States to investigate how priming effect of the media affects their attitudes about four different countries. Willnat et al. (2000) also pointed out the reports that initiated terrorism or drugs significantly affected the attitudes of the participants toward Mexico and Colombia. Matthes and Beyer (2017) used a theoretical cognitive-affective process model of the hostile media effect (HME) to prove that the perception of media of people itself may also affect their attitudes toward a certain issue. In other words, media not only have the effect of initiating the attention of people to a certain topic but also have the possibility of inspiring people to have positive or negative effects on a certain topic due to cognitive tendency of people to media and may even cause adverse effects. It fully indicates the complicated mechanism of influence of media on attitudes of individuals on other countries.

The second function of the media is the framing function, which is closely related to the agenda-setting function of the media (Moy et al., 2016). Price et al. (1997) pointed out that frames can affect cognitive and affective perceptions of readers of a story. Based on reviewing the research on the role of media in the framework, Dell'Orto et al. (2004) examined how the democratic and non-democratic frameworks of the country in American newspapers affect perceptions and images of foreign countries in readers. Ospina Estupinan (2017) confirms that countries in Latin America do have a typical framework for international image of China. In government evaluation, this kind of framing function of news media still exists. For example, Shen and Guo (2013) indicate that the information frame in reports tend to activate the relevant psychology of the public in political evaluation. Zhao et al. (1994) found that the use of news media is positively related to government policy support. In other words, in response to the evaluation of government of China, the media frame that the Chinese public is exposed to may strengthen their positive attitude toward the government. What about other countries? Will the reporting frame distributed in different media affect Chinese perception of controlling-pandemic evaluations of other countries?

Indeed, the influence of media on government evaluation is reflected in the most basic level of information acquisition. For example, Wanta et al. (2004) confirmed that the more media reports focus on one country, the more the public thinks the country is important. If they receive more negative information about a country, they are more likely to have a bad impression of the country. Lee and Hong (2012) also confirmed this view with data from 27 countries. Furthermore, we should also pay attention to the influence of the own frame of the media on the perception of government evaluation. For example, Shen and Guo (2013) proved that the internet is strongly negatively correlated with political trust through world values data, whereas TV news and political trust are significantly and positively correlated. On the other hand, the influence of newspapers is not significant. Shen et al. research is rooted in his assumption that many Chinese media are propaganda. So when reporting frame of media or political attributes are linked to the evaluation of foreign governments, what will happen?

Under the premise that media may have an impact on controlling-pandemic evaluations by foreign governments, we also need to consider the influence of bias of different media toward controlling-pandemic reports on other countries. For example, Shen and Guo (2013) indicated that television and political trust of Chinese citizens on the government is significantly and positively correlated, while the internet is the opposite. A large number of studies also indicated the negative correlation that the rise of the internet may have on the evaluation of the Chinese government (Yang, 2003). So when the internet gradually becomes old-fashioned, will newer forms of media, such as social media, have more influence on government evaluations and evaluations of other countries? In China during the pandemic, social media functioned as an intermediary and filtered other media information releases. It has become a comprehensive information exposure method facilitating media contact and interpersonal contact. Then, whether does the information circulated on different social media has a differential impact on evaluation of different countries by people? Han and Xu (2020) have shown that social media has played a more important role than traditional media in improving public health. In the evaluation of governments of other countries, we have reason to speculate that the use of different media will have different effects, so we propose the following:

H3. The exposure of Chinese public to different media types will affect their evaluation of the performance of governments in controlling the pandemic.

The factors that are generally influencing are as follows: socio-demographic variables, virus perception, and cultural value orientation; in addition to the above three main factors, we will also consider the possible impact of general demographic variables, virus perception, and cultural value orientation on government evaluation. Demographic variables are the basic variables when we examine various government evaluations, and the current government performance evaluation from the citizen perspective further magnifies the significance of demographic variables (Alshawi and Alalwany, 2009). Among them, we pay special attention to the influence of political parties on the evaluation of the government. Bian et al. (2001) confirmed that the attributes of party members are closely related to the dynamics of the system, and the attributes of individual party affiliations are very likely to affect their perception of other countries. Cultural values have a similar influence. Collectivist cultural values are considered to be east Asian and are also considered as an important variable when analyzing various political phenomena in China (Shi, 2001; Yang et al., 2014). Dahler-Larsen and Schwandt (2012) pointed out that understanding government evaluation must be based on the political culture of the country, so we incorporate collectivist cultural values as a factor. In addition, the impact of the evaluation of the fight of the government against the pandemic will also be related to perception of the virus itself by people. If a higher awareness of the danger of the virus is held, will it reduce the strict judgments of the public on the governments of various countries? Or is it that the higher the awareness of the danger of the virus, the more we hope that countries can control it and increase expectations? Therefore, we incorporate the above three types of variables into the overall analysis framework and finally form a three-dimensional model based on cognition-emotion-media communication.

### Aim

This research aims to examine the explanatory power of the cognitive-affective model for the evaluation of the performance of other countries in controlling COVID-19 by the Chinese public, and to analyze the role of media use in it, and to construct a three-dimensional model of cognitive-affective-media.

## Methods

### Participants

The data of this study comes from a random sampling survey conducted on the Chinese large-scale questionnaire survey platform (https://www.wjx.cn/) from June 3–7, 2020. The survey took 2.6 million registered users as the sample pool. A total of 1,358 questionnaires were distributed through continuous rolling random questionnaires, of which 619 were valid (the rate of valid was 45.58%). Because the survey was continuously distributed randomly, so the questionnaire obtained still meets the requirements. The survey also uses IP address and logic problem design to ensure that each participant only participates in the survey one time. The population covered by the survey involved a total of 30 provinces, municipalities, and autonomous regions across the country (the Tibet autonomous region did not collect samples).

The demographic characteristics of the sample are distributed as shown in Table 1:

**Table 1:** Distribution of sample socio-demographics.

	**Categories**	**Frequency**	**Percentage (%)**		**Categories**	**Frequency**	**Percentage (%)**
Gender	Male	359	58.0	Province or municipality	Hebei	56	9.0
	Female	260	42.0		Hubei	53	8.6
Education	Junior high school and below High school	11	1.8		Guangdong	46	7.4
		25	4.0				
	College/University	514	83.0		Shanghai	46	7.4
	Master and above	69	11.2		Liaoning	35	5.7
Family income per	<4,999	65	10.5		Shanxi	32	5.2
Month (Rmb)	5,000–9,999	166	26.8		Zhejiang	32	5.2
	10,000–14,999	161	26.0		Hunan	30	4.8
	15,000–19,999	127	20.5				
	20,000–24,999	58	9.4		Henan	29	4.7
	>25,000	42	6.8		Jiangsu	27	4.4
	City	464	75.0		Fujian	25	4.0
	Town	97	15.8		Beijing	22	3.6
	Rural	58	9.2		Tianjin	20	3.2
Party member	CCP	125	20.2		Chongqing	19	3.1
	Non-CCP	494	79.8		Sichuan	18	2.9
Age	Mean	30.5			Others	129	20.8

### Measures

#### The Evaluation of COVID-19 Control by Different National Governments

The dependent variable of this study is the evaluation of the controlling-pandemic performance of the governments of different countries by the Chinese public. Although the government evaluation itself has many dimensions, according to research needs we mainly adopt the 0–10 points scoring system (Sanderson, 2001). The specific questions are as follows: “Please rate the controlling-pandemic situation of the following countries (0 is very poor, 10 is very good).” The matrix is scored for 12 countries. Refer to Table 2 for the mean and SD of scores by the countries.

**Table 2:** Evaluation of controlling-pandemic, pandemic perception, and feeling thermometer of the Chinese public in different countries (*N* = 619).

	**Evaluation of controlling-pandemic (0–10)**		**Pandemic perception (1–5)**		**Feeling thermometer (1–5)**	
	**Mean**	**Std**.	**Mean**	**Std**.	**Mean**	**Std**.
US	**1.42**	1.78	**4.94**	0.28	**1.69**	0.90
Japan	5.57	2.08	3.53	0.70	**2.40**	1.01
UK	3.97	1.92	**4.14**	0.71	2.67	0.83
S.Korea	6.02	2.02	3.46	0.79	2.79	0.90
Italy	4.76	2.11	**4.44**	0.71	2.97	0.92
Germany	5.39	1.99	3.74	0.78	3.26	0.86
France	4.95	1.87	3.83	0.79	3.17	0.90
Iran	4.84	1.89	3.87	0.85	2.91	0.85
Brazil	**3.84**	2.16	4.10	0.84	2.82	0.84
India	**3.55**	2.16	4.07	0.92	**2.07**	0.89
Russia	5.48	2.12	3.86	0.94	3.68	0.87
China	9.10	1.35	3.27	0.93	4.84	0.59

*The bold values are the values of the top three countries in each index*.

#### Pandemic Perception of Different Countries

Regarding the public perception of the epidemic situation in different countries, similar to Jose's seven-point ranking scale (Jose et al., 2021) on the epidemic perception, we used the following five-degree Likert scale to measure according to the actual measurement purpose. It is “How do you perceive the seriousness of the spread of COVID-19 in the following countries?” The answers are 1 = very serious, 2 = relatively serious, 3 = fair, 4 = relatively slight, and 5 = slight. After the reverse assignment, the average and SD of the scores on this indicator of the Chinese public are shown in Table 2.

#### Feeling Temperature of Different Countries

**Figure 1:**
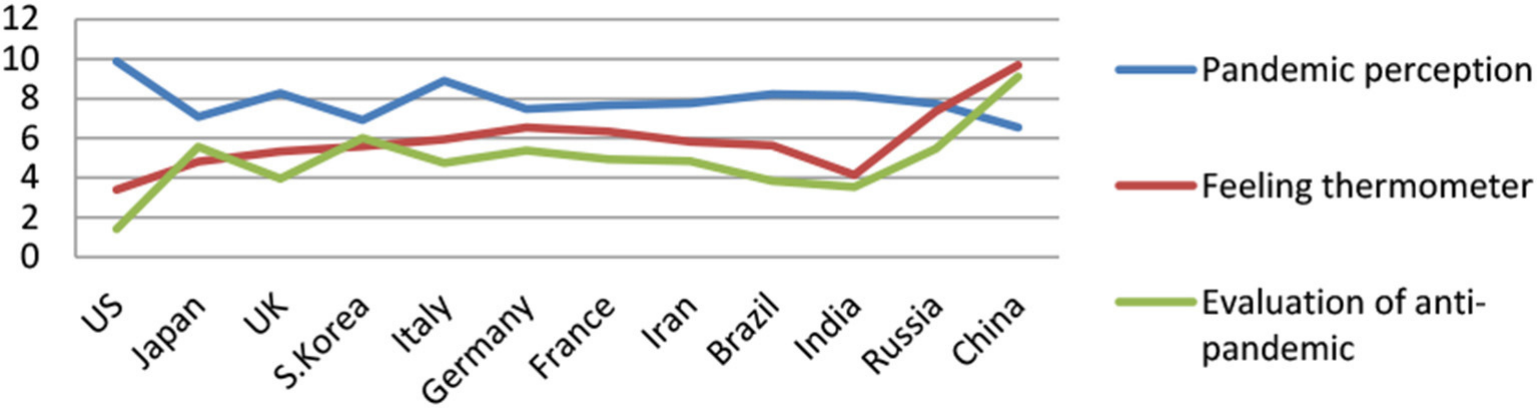
Evaluation of controlling-pandemic, feeling thermometer and pandemic perception of Chinese public on different countries (*N* = 619).

There are various ways to measure feeling temperature (Liu and Wang, 2015). The most common one is a 0–100 scoring system (Greene, 2004). To facilitate respondents to answer questions more quickly, the following question is used to measure: “How do you like or dislike the following countries,” countries include the 12 countries shown in Table 2, and the answers are 1 = like it very much, 2 = like it more, 3 = fair, 4 = I do not like it, and 5 = I do not like it very much. After the reverse assignment, the average and SD of the feeling temperature of the Chinese public for each country are shown in Table 2. This item is used to measure national feeling, and, sometimes, it is directly replaced with the feeling thermometer (refer to Table 2 and Figure 1).

#### Media Exposure for COVID-19

The third influencing variable examined in this study is the media exposure of Chinese residents to pandemic information in various countries. Considering the history of media development and actual media exposure of residents in China, media exposure is mainly divided into two categories: exposure to pandemic information through traditional media and exposure to pandemic information through social media. Traditional media include newspapers, magazines, broadcast, television, and the internet. Social media include WeChat, Weibo, Tiktok, Kuaishou, QQ, BaiduTieba, Zhihu, Douban, Facebook, Twitter, and Instagram (Han and Xu, 2020). The specific measurement question is “How do you receive various pandemic information (including the number of infections, global spread, prevention methods, discussion of different viewpoints, etc.) from the following media,” the answers are 1 = very more, 2 = more, 3 = general, 4 = less, and 5=less or no, and the score is reversed. After re-assignment, the minimum value is 1, and the maximum value is five, the average value of each media and SDs are given for newspapers (M = 1.82 and SD = 0.98), magazines (M = 1.69 and SD = 0.88), broadcast (M = 4.21 and SD =0.77), Weibo (M = 3.50 and SD = 1.24), Tiktok (M = 3.28 and SD = 1.28), Kuaishou (M = 2.63 and SD = 1.34), QQ (M = 2.95 and SD = 1.12), BaiduTieba (M = 2.53 and SD = 1.14), Zhihu (M = 2.64 and SD = 1.20), Douban (M = 1.98 and SD = 1.02), Facebook (M = 1.45 and SD = 0.81), Twitter (M = 1.44 and SD = 0.82), and Instagram (M = 1.39 and SD = 0.78).

### Measurement of Other General Variables That Affect Government Evaluation

#### COVID-19 Perception

Since there is no mature scale on COVID-19 Perception to be adopted, we have conducted multiple rounds of question screening through consulting experts and official science information of WHO to determine the following measurement question. It is “Please express your attitude toward the following statements about COVID-19.” The measurement items include eight statements: (1) the new coronavirus is more harmful than the SARS virus; (2) at present, we have a deeper understanding ofCOVID-19; (3) people infected with COVID-19 can be cured; (4) the death rate among patients infected by COVID-19 is very high; (5) the latent impact of COVID-19 is very large; (6) the COVID-19 pandemic can be completely controlled in our country after 6 months; (7) the COVID-19 pandemic can be controlled globally in the next year; and (8) there will be repeated outbreaks of COVID-19 in the future. The options are: 1 = strongly agree; 2 = more agree; 3 = general, 4 = more disagree, and 5 = strongly disagree, where 1.4.5.8 reverse scoring is used. After the weighted summary, the copy range of this item is 1–5. The average value of the variable is 3.16, the SD is 0.44, and Cronbach's Alpha is 0.735. The KMO value is 0.715, and the Bartlett sphere test result is significant (0.00).

#### Collectivist Cultural Value

With reference to the measurement method of the Asian Barometer Survey (Han et al., 2011), the measurement issues of this study include the following: (1) the state is like a big machine, and the individual is but a small cog, with no independent status. (2) Personal interests give way to collective interests, in general. (3) Personal interests should be sacrificed for national interests. (4) For the benefit of the family, individuals should put their interests second. (5) Even if request of a parent is unreasonable, children should still obey. (6) If a conflict occurs, one should ask senior people to uphold justice. The options are as follows: 1 = strongly agree; 2 = more agree; 3 = general, 4 = more disagree, and 5 = strongly disagree, and the indicators of collectivist cultural value are obtained after reverse scoring and total average. The average value of this variable is 3.12, the SD is 0.56, and Cronbach's Alpha is 0.705. The KMO value is 0.694, and the Bartlett sphere test result is significant (0.00).

#### Demographic Characteristics

The demographic variables used in this study mainly include gender, age, education, income, and party affiliation. The operational measurement of each indicator is as follows: gender, 1 = male; 0 = female and the female was the control group. Age: calculated using 2020 minus the year of birth. Education level: 1 = junior high school and below; 2 = high school/secondary school, technical school; 3 = college, university; 4 = Master; and 5 = PhD and above. The family income per month: 1 = 4,999 Yuan or less, 2 = 5,000–9,999 Yuan, 3 = 10,000–14,999 Yuan, 4 = 15,000–19,999 Yuan, 5 = 20,000–24,999 Yuan, 6 = 25,000 Yuan or more. Party: 1 = CCP and 2 = Non-CCP.

#### Data Analysis Methods and Procedures

According to the research hypothesis, we used the evaluation of the controlling-pandemic performance of the governments of different countries by the Chinese public as the dependent variable, and the relevant influencing variable as the independent variable, and processed the data through multiple regressions (OLS regression in SPSS19.0 software). Independent variables in the model are mainly composed of four categories: demographic variables, traditional media information exposure, social media information exposure, and perception of different countries (pandemic perception and feeling temperature). We believe that in this way, we can observe the distribution of the influence of different variables in the evaluation of pandemic governance in various countries, and from this, we can also discover which factors are the most important and have common effects.

## Results

### Descriptive Results of Controlling-Pandemic Evaluation, Pandemic Perception, and Feeling Temperature in Different Countries by Chinese Public

Based on the 619 data samples, the top three perception of the pandemic by the Chinese citizens are the United States, Italy, and the United Kingdom, and the bottom three in the emotional score are the United States, India, and Japan, the last three in controlling-pandemic performance are the United States, India, and Brazil. The results of pairwise *t*-test and Bootstrap analysis show that the evaluation of different countries by the Chinese public in various categories differs significantly (*p* < 0.05). Refer to Table 2 for details.

To better show the performance of the Chinese citizens in handling the pandemic in different countries, the severity of the pandemic and the feeling temperature, after the perception of the pandemic and the feeling temperature performance are all converted into a 0–10 measurement (original value ^*^2), Figure 1 is obtained as follows.

It can be seen from Figure 1 that the controlling-pandemic evaluation and the trend of feeling temperature changes are the same, showing a positive correlation; while it is opposite to the trend of pandemic perception, showing a negative correlation. Chinese people have the lowest feeling temperature toward the United States, the highest perception of the pandemic, and the lowest controlling-pandemic evaluation, whereas they have the highest feeling temperature toward China, lower perception of the pandemic, and the highest controlling-pandemic evaluation.

### Analysis of Regression Results

It can be seen from Table 3 that variables selected by this study can explain the changes in evaluation of the Chinese citizens of the fight among different countries against the pandemic. All regression equations are significant. The lowest adjusted *R*^2^ coefficient is the French regression model (Adjusted *R*^2^ = 0.089), the highest is the Brazil regression model (Adjusted *R*^2^ = 0.218), and the significance of each regression model is 0.000. By further observing the explanatory power of different influencing variables in each equation, it can be found that feeling temperature and pandemic perception, in general, have influence, with a significant degree of 0.00 (only in the Iran regression equation model, pandemic perception has no significant influence) and the explanatory coefficients in each equation are far higher than other influencing factors. Pandemic perception is negatively correlated with controlling-pandemic evaluation, whereas national feeling temperature is positively correlated with controlling-pandemic evaluation. These results support H1 and H2.

**Table 3:** Regression analysis for evaluation of controlling-pandemic of the Chinese public in different countries.

		**M1**	**M2**	**M3**	**M4**	**M5**	**M6**	**M7**	**M8**	**M9**	**M10**	**M11**	**M12**
		**US**	**Japan**	**UK**	**S.Korea**	**Italy**	**Germany**	**France**	**Iran**	**Brazil**	**India**	**Russia**	**China**
		**B(β)**	**B(β)**	**B(β)**	**B(β)**	**B(β)**	**B(β)**	**B(β)**	**B(β)**	**B(β)**	**B(β)**	**B(β)**	**B(β)**
Demography variables	Gender	−0.115 (0.032)	0.095 (0.022)	0.202 (0.052)	0.244 (0.060)	0.254 (0.059)	0.353^*^ **(0.087)**	0.155 (0.041)	0.018 (0.005)	−0.072 (−0.016)	0.375^*^ **(0.086)**	0.169 (0.039)	−0.097 (−0.035)
	Age	0.015 (0.072)	0.019 (0.075)	0.007 (0.032)	0.039 (0.163)	0.012 (0.047)	0.019 (0.080)	0.018 (0.080)	0.004 (0.018)	0.005 (0.018)	0.009 (0.035)	−0.013 (−0.053)	−0.016^*^ **(−0.103)**
	Education	−0.318^*^ **(−0.085)**	−0.051 (−0.012)	−0.232 (−0.057)	0.263^****^ **(0.062)**	−0.197 (−0.044)	0.183 (0.044)	−0.057^*^ **(−0.015)**	−0.173 (−0.044)	−0.044 (−0.010)	−0.035 (−0.008)	−0.044 (−0.010)	−0.230 (−0.081)
	Income	−0.071 (−0.054)	0.200**^*^ **(0.131)**	−0.108 (−0.076)	0.104 (0.070)	−0.085 (−0.055)	−0.032 (−0.022)	−0.114 (−0.084)	0.047 (0.034)	−0.109 (−0.069)	−0.079 (−0.050)	−0.029 (−0.019)	0.073 (0.073)
	MCP	0.287 (0.064)	−0.044 (−0.009)	−0.049 (−0.010)	−0.090 (−0.018)	−0.397 (−0.075)	−0.144 (−0.029)	−0.167 (−0.036)	0.223 (0.047)	0.476^*^ **(0.088)**	0.212 (0.039)	−0.062 (−0.012)	−0.168 (−0.050)
Control variables	Culture Collectivism	−0.059 (−0.018)	−0.138 (−0.037)	−0.050 (−0.014)	−0.264 (−0.073)	0.081 (0.021)	−0.164 (−0.046)	−0.184 (−0.055)	−0.157 (−0.047)	−0.155 (−0.040)	−0.126 (−0.033)	−0.159 (−0.042)	0.069 (0.028)
	COVID−19 Perception	−0.323^*^ **(−0.079)**	0.017 (0.003)	−0.215 (−0.049)	−0.182 (−0.040)	−0.382^*^ **(−0.079)**	0.039 (0.009)	−0.098 (−0.023)	0.059 (0.014)	−0.225 (−0.045)	−0.102 (−0.021)	0.098 (0.020)	−0.043 –(0.014)
Traditional media	newspaper	−0.017 (−0.009)	−0.269^**^ **(−0.126)**	−0.177 (−0.090)	−0.052 (−0.025)	0.059 (0.027)	−0.023 (−0.011)	0.040 (0.021)	0.116 (0.060)	−0.166 (−0.075)	−0.062 (−0.028)	0.026 (0.012)	−0.093 (−0.068)
	Magazine	0.131 (0.064)	0.014 (0.006)	0.200 (0.091)	−0.150 (−0.065)	0.116 (0.048)	−0.019 (−0.008)	0.031 (0.014)	−0.137 (−0.064)	0.264^*^ **(0.107)**	0.097 (0.040)	0.088 (0.037)	0.165 (0.107)
	broadcast	0.002 (0.002)	0.049 (0.028)	0.022 (0.013)	−0.016 (−0.010)	−0.152 (−0.083)	−0.100 (−0.058)	−0.018 (−0.011)	−0.034 (−0.021)	0.023 (0.013)	−0.072 (−0.039)	−0.041 (−0.023)	0.028 (0.024)
	TV	−0.211^*^ **(−0.110)**	0.108 (0.048)	−0.034 (−0.017)	0.159 (0.073)	0.004 (0.002)	0.226^*^ **(0.105**)	0.005 (0.002)	0.160 (0.078)	−0.072 (−0.031)	0.109 (0.047)	0.083 (0.036)	0.151^*^ **(0.103)**
	Internet	−0.055 (−0.019)	0.226 (0.067)	0.070 (0.022)	0.282 (0.086)	−0.041 (−0.012)	0.009 (0.003)	0.025 (0.008)	0.175 (0.057)	0.205 (0.059)	−0.022 (−0.006)	0.037 (0.011)	−0.054 (−0.025)
Social media	WeChat	0.022 (0.010)	−0.096 (−0.036)	−0.106 (−0.043)	−0.291^**^ **(−0.113)**	0.038 (0.014)	−0.143 (−0.056)	−0.179 (−0.075)	−0.217^*^ (−0.090)	−0.061 (−0.022)	−0.057 (−0.021)	−0.108 (−0.040)	−0.060 (−0.035)
	Weibo	−0.033 (−0.023)	−0.072 (−0.043)	0.028 (0.018)	0.065 (0.040)	0.055 (0.032)	−0.033 (−0.021)	0.004 (0.003)	−0.038 (−0.025)	0.028 (0.016)	0.092 (0.053)	−0.191^*^ **(−0.111)**	−0.033 (−0.030)
	Tiktok	0.003 (0.002)	−0.107 (−0.066)	0.077 (0.051)	−0.124 (−0.079)	0.096 (0.058)	0.061 (0.039)	0.089 (0.061)	0.008 (0.005)	0.064 (0.038)	0.142^*^ (0.084)	0.148^*^ **(0.089)**	−0.014 (−0.013)
	Kuaishou	0.106 (0.079)	0.075 (0.048)	−0.044 (−0.031)	0.042 (0.028)	−0.157^*^ **(−0.099)**	−0.116 (−0.078)	−0.085 (−0.061)	−0.064 (−0.045)	−0.042 (−0.026)	−0.092 (−0.057)	−0.094 (−0.059)	−0.050 (−0.049)
	QQ	0.016 (0.010)	0.086 (0.046)	0.080 (0.046)	0.140 (0.078)	0.159 (0.084)	0.011 (0.006)	0.030 (0.018)	0.128 (0.076)	0.033 (0.017)	0.143 (0.074)	0.064 (0.034)	0.033 (0.027)
	BaiduTieba	0.083 (0.053)	−0.019 (−0.010)	−0.032 (−0.019)	−0.044 (−0.025)	−0.078 (−0.042)	0.055 (0.031)	0.080 (0.049)	−0.060 (−0.036)	−0.011 (−0.006)	−0.034 (−0.018)	−0.028 (−0.015)	0.063 (0.053)
	Zhihu	0.141^*^ **(0.095)**	0.086 (0.049)	0.101 (0.063)	0.149 (0.089)	0.070 (0.040)	0.127 (0.077)	0.118 (0.076)	0.023 (0.015)	0.076 (0.042)	−0.079 (−0.044)	0.034 (0.019)	−0.039 (−0.035)
	Douban	−0.051 (−0.029)	0.014 (0.007)	0.020 (0.011)	−0.081 (−0.041)	0.189 (0.090)	−0.011 (−0.005)	−0.025 (−0.014)	−0.052 (−0.028)	−0.146 (−0.068)	0.038 (0.018)	0.022 (0.010)	0.039 (0.029)
	Facebook	0.127 (0.058)	−0.116 (−0.045)	−0.016 (−0.007)	0.022 (0.009)	0.097 (0.037)	−0.064 (−0.026)	−0.018 (−0.008)	−0.003 (−0.001)	0.286 (0.107)	0.162 (0.061)	0.092 (0.035)	−0.244^*^ **(−0.146)**
	Twitter	0.040 (0.018)	0.083 (0.032)	0.118 (0.050)	−0.120 (−0.049)	0.039 (0.015)	0.159 (0.065)	0.243^*^ (0.107)	0.238 (0.103)	−0.013 (−0.005)	0.207 (0.079)	0.256 (0.099)	0.014 (0.009)
	Instagram.	−0.209 (−0.091)	−0.025 (−0.009)	0.112 (0.045)	0.053 (0.020)	−0.039 (−0.014)	−0.038 (−0.015)	−0.088 (−0.037)	0.007 (0.003)	−0.032 (−0.012)	−0.153 (−0.055)	−0.140 (−0.051)	−0.038 (−0.022)
Perception on different country	Pandemic perception	−1.056^****^ **(−0.171)**	−0.782^****^ **(−0.265)**	−0.698^****^ **(−0.258)**	−0.613^****^ **(−0.240)**	−0.385^****^ **(−0.129)**	−0.511^****^ **(−0.202)**	−0.449^****^ **(−0.192)**	−0.143 (−0.065)	−0.975^****^ **(−0.382)**	−0.692^****^ **(−0.297)**	−0.665^****^ **(−0.297)**	−0.161^**^ **(−0.111)**
	Feeling temperature	0.383^****^ **(0.194)**	0.477^****^ **(0.232)**	0.337^****^ **(0.146)**	0.331^****^ **(0.149)**	0.377^****^ **(0.164)**	0.592^****^ **(0.256)**	0.352^****^ **(0.171)**	0.641^****^ **(0.290)**	0.347^****^ **(0.135)**	0.591^****^ **(0.244)**	0.531^****^ **(0.219)**	0.494^****^ **(0.217)**
	F	4.538^****^	5.783^****^	4.764^****^	4.373^****^	2.981^****^	4.865^****^	3.414^****^	3.249^****^	7.888^****^	6.434^****^	5.848^****^	3.178^****^
	Adjusted *R*^2^	0125	0.162	0.132	0.120	0.074	0.135	0.089	0.083	0.218	0.180	0.164	0.081
	*R*^2^	0.161	0.192	0.167	0.156	0.122	0.170	0.126	0.120	0.250	0.213	0.198	0.118

* *p ≦ 0.05*,

** *p≦ 0.01*,

*** *p≦ 0.001*,

**** *p = 0.000*.

In terms of media exposure and national performance evaluation, different countries show different distribution characteristics. Specifically, the forms of media that has a significant impact on the evaluation of the United States controlling-pandemic are TV (β = −0.110, *p* < 0.05) and Zhihu (β = 0.095, *p* < 0.05); the form of media that has a significant impact on the evaluation of Japan is newspapers (β = 0.126, *p* < 0.01); the form of media that has a significant impact on the evaluation of South Korea is WeChat (β = −0.113, *p* < 0.01); the form of media that has a significant impact on Italy is Kuaishou (β = 0.099, *p* < 0.05); for Germany, TV has a significant effect (β = 0.105, *p* < 0.05); magazine has a significant effect on Brazil (β = 0.1079, *p* < 0.05); the form of media that has a significant effect on India is Tiktok (β = 0.084, *p* < 0.05); the forms of media that have a significant impact on Russa are Weibo (β = −0.111, *p* < 0.05) and Tiktok (β = 0.148, *p* < 0.05); the forms of media that have a significant impact on China are TV (β = 0.103, *p* < 0.05) and Facebook (β = −0.146, *p* < 0.05). These partially support H3. It also explains the difference in the evaluation of controlling-pandemic effects in various countries by the media.

In terms of the impact of general variables on the evaluation of the national controlling-pandemic performance, we found that COVID-19 perception has a significant impact on the evaluation of government controlling-pandemic performance in the United States (β = −0.079, *p* < 0.05) and Italy (β = 0.079, *p* < 0.05). Collectivist cultural value, in general, has no significant influence. Among them, CCP members rated the (β = 0.088, *p* < 0.05) controlling-pandemic performance of Brazil government more positively, people with high incomes rated Japan more positively (β = 0.131, *p* < 0.001), and people with high education rated South Korea (β = 0.062, *p* < 0.05) more positively, while rated the United States (β = −0.085, *p* < 0.05) and France (β = −0.015, *p* < 0.05) more negatively. Older people are negatively correlated with the performance evaluation of China (β = −0.103, *p* < 0.05), while men have more positive evaluations of controlling-pandemic in Germany (β = 0.087, *p* < 0.05) and India (β = 0.086, *p* < 0.05).

## Conclusion and Discussion

This study mainly discusses the impact of pandemic perception, national feeling, and media use on evaluation of controlling-pandemic performance in different countries from the three-dimensional perspective of cognitive-affective-media by Chinese residents. The results show the following: (1) pandemic perception and feeling temperature are important factors that affect the evaluation of controlling-pandemic performance in different countries by Chinese public. Pandemic perception is negatively correlated with controlling-pandemic performance evaluation, while feeling temperature is positively correlated with controlling-pandemic performance evaluation. (2) The use of media has different characteristics in the evaluation of the controlling-pandemic performance of different countries by Chinese public. Among them, television has played a significant role in the evaluation of the controlling-pandemic performance by Chinese public in the United States, China, and Germany. This is likely because television reports have a special focus on these countries. (3) Cultural orientation has no significant impact on the evaluation of controlling-pandemic performance in different countries by Chinese public, and COVID-19 perception only has a significant impact on the evaluation of controlling-pandemic performance in some countries (the United States and Italy). Demographic variables have different characteristics for the public to evaluate the controlling-pandemic performance of different countries.

As a descriptive study of a small sample, the above findings provide basic information to understand the evaluation of controlling-pandemic evaluations by Chinese public and influencing factors in different countries during this pandemic, but what is the significance of these findings? Is the three-dimensional model sufficient for the evaluation by the Chinese public of the government of another country? The three, namely the cognitive-affective-media factors we propose, are there more complex relationships among each other? Regarding the existing research, what tentative exploration did this research make? Reviewing the reasons for the research, research design and previous research literature, this article mainly hopes to make verification and contributions in the following two theoretical construction directions.

### In the Evaluation of Other Governments, There Is a Cognitive-Affective Model, That Is, Cognition and Emotion Play a Dual Role

The cognitive-affective model is widely used in tourism destination management and national and local image research; however, on the issue of government evaluation, people have always focused on the influencing factors of a country's people's evaluation of their own government, especially after the rise of government performance evaluation and NPM (Hood, 1991)Attention is often focused on structural factors in national governance, such as elections and bureaucracy, process factors, such as citizen participation, and result factors, such as corruption governance and citizen satisfaction (Xiao and Xiao, 2016). These factors are not enough for people in other countries to measure the effectiveness of a government. In other words, from the perspective of the “other,” some deep-seated factors that initiate cognition are playing a role. Dryhurst et al. (2020) pointed out that individualistic worldviews, personal experience, and prosocial values will all play a role in the formation of risk perception of people on COVID-19 in various countries. In addition to factors, which play a role, whether there is a basic cognition formation structure determines attitudes and views of people. Indeed, in recent years, the research background of affective factors entering the field of government evaluation has provided an inspiration. Different researchers have paid enough attention to affection in government research from the micro, meso, and macro levels. For example, Ennis et al. (2018) examined the role of emotional and normative commitments in turnover intentions of government employees at the micro-level. Wilson (2015) and Miller et al. (2004) examined intergroup emotions as an important mediator between intergroup contact and general political predispositions. At the macro level, a study of Twitter information about China shows that when non-negative emotional information about pandemic control decreases in China, discussions about Chinese politics, diplomacy, and racist ideology arise. It indicates that emotional comments on political information may have a greater influence than the long-term impact of cognitive information (Chen et al., 2020). These research cases provide an opportunity to use the “cognitive-affective” two-factor model to analyze evaluation of the performance of governments of other countries by the Chinese public during the COVID-19 pandemic. This research also shows a strong correlation between the two factors and country evaluation.

### The Media Plays a Differentiated Role in the Evaluation of Governments of Other Countries, Which Can Verify the Cognitive-Affective-Media Attitude Formation Mechanism, but the Enhancement or Amplification Effect of the Media on Cognition and Affection Needs Further Research

Existing studies have shown that the priming function (Iyengar and Kinder, 1987; Miller and Krosnick, 2000; Willnat et al., 2000; Brewer et al., 2003; McGraw and Ling, 2003; Matthes and Beyer, 2017), framing function (Zhao et al., 1994; Price et al., 1997; Dell'Orto et al., 2004; Shen and Guo, 2013; Moy et al., 2016; Ospina Estupinan, 2017), information function (Wanta et al., 2004; Lee and Hong, 2012; Shen and Guo, 2013) of the media are used in government evaluation. At the same time, the media itself also has a bias (Yang, 2003; Shen and Guo, 2013) and substitution effects (Kaye and Johnson, 2003; Han and Xu, 2020). This research further demonstrates how the potential attributes of different media influence the evaluation of the governance of different countries by the Chinese public in the control and management of the COVID-19 pandemic. In the end, we saw that TV, as a mainstream media, under the enhanced effect of home isolation measures during the pandemic, the strengthening of the positive evaluation of the Chinese government, and the strengthening of the negative evaluation of the United States government occurred. Meanwhile, whether it is traditional media or social media, the influence of different media on the evaluation of governments of different countries is different, which means that there is a media map in the evaluation of governments of other countries. Understanding the reporting bias, coverage, and audience distribution of various media in a country is very useful for predicting the national evaluation of the public. It should be noted that, when social media information is easy to offset each other and polarization exists at the same time, Chinese TV still plays a leading role in public opinion.

Regarding the superimposing effect of media in public cognition and affection, Ball-Rokeach and DeFleur (1976) analyzed the possible influence of media on public cognition, affection, and behavior. It is called the dependency model of media effects. Further, Kepplinger et al. (1991) proposed cognitive-affective media effects, and also developed a theoretical cognitive-affective process model of the HME. These studies show that the media influence the emotions and cognition of audience. In this research, affection and cognition are variables that have been extracted separately, so is there a superimposed effect of media on cognition and affection, that is, in specific events, the media will further exert its influence on cognition and affection, forming a superimposed effect model of cognition-emotion + media? This becomes the direction of the subsequent expansibility research.

### Limitations

This study is an analysis of the general factors that affect evaluation of Chinese citizens of the anti-pandemic performance of different countries, with a particular focus on the impact of pandemic perception, national feeling, and media use. Although this framework attempts to integrate the cognitive and affective perspectives that affect the formation of national attitudes and highlights the role of media, there is no further analysis of impact of media on the perception of the pandemic and national feeling, so the results of the regression analysis appear to be relatively flat. It is worthwhile to investigate the strengthening/weakening influence of media in the formation of cognitive and emotional functions through structural equation analysis. We also examine other factors including gender, age, education, income, political party, cultural collectivism, and COVID-19 perception. Among them, cultural collectivism is related to affection, and COVID-19 perception is related to cognition. The selection of these variables may appear relatively arbitrary, and there is no in-depth analysis of the research results, such as why cultural collectivism does not work in general, and COVID-19 perception only plays a role in the evaluation of the United States and Italy. However, this uncontrolled regression analysis method is helpful to show the true status of evaluation of Chinese residents on the pandemic control in different countries. We also hypothesize that it is more of a propensity analysis than rigorous causal analysis (Rubin and Waterman, 2006). In addition, in the measurement of media use, traditional media includes the internet based on portal website use, and the specific classification of social media into 11 categories is likely to cause doubts about the levels of measurement; however, this categorization is carried out under the guidance of previous research (Ho et al., 2015; Han and Xu, 2020) and the intention to examine the comparative effects of social media and traditional media. The research conducted a dialogue on the classification of media use. This research presents the main influencing factors of the evaluation of the anti-pandemic by the Chinese public in different countries during the COVID-19 pandemic. The perception of the pandemic and national feeling plays a dual role, and media exposure has a differentiated and diverse influence on this kind of evaluation. In China, TV still exerts an important influence on the evaluation of major countries. These study results also provide reflections on how to eliminate barriers between different countries and face human disasters together in the context of the global public health crisis.

## Data Availability Statement

The raw data supporting the conclusions of this article will be made available by the authors, without undue reservation.

## Ethics Statement

The studies involving human participants were reviewed and approved by ethics committee of School of Media and Communication, Shanghai Jiao Tong University. Written informed consent to participate in this study was provided by the participants in Wenjuanxing survey platform (https://www.wjx.cn/).

## Author Contributions

RH: conceptualization, data curation, formal analysis, methodology, and writing—original draft. RH and JX: methodology, funding acquisition, investigation, and writing— review and editing. JX: supervision. All authors contributed to the article and approved the submitted version.

## Conflict of Interest

The authors declare that the research was conducted in the absence of any commercial or financial relationships that could be construed as a potential conflict of interest.
